# Diagnostic Tips from a Video Series and Literature Review of Patients with Late-Onset Tay-Sachs Disease

**DOI:** 10.5334/tohm.726

**Published:** 2022-12-27

**Authors:** Giulietta Maria Riboldi, Heather Lau

**Affiliations:** 1 The Marlene and Paolo Fresco Institute for Parkinson’s Disease and Movement Disorders, New York University Langone Health, New York, NY, United States of America; 2 Department of Neurology, New York University Langone Health, New York, NY, United States of America; 3 Department of Internal Medicine, Yale University, New Haven Connecticut, United States of America

**Keywords:** Late-Onset Tay-Sachs, hexosaminidase enzyme, cerebellar, motoneuron, psychiatric, stuttering, diagnostic tips

## Abstract

**Background:**

Late-Onset Tay-Sachs (LOTS) disease is a rare, progressive neurological condition that can dramatically affect the life of these patients. The diagnosis of LOTS is easily missed because of the multifaced presentation of these patients, who can initially be assessed by neuromuscular or movement disorder specialists, or psychiatrists. Clinical trials are now becoming available for LOTS. Therefore, early diagnosis can be detrimental for these patients and for insuring informative research outcomes.

**Methods:**

We characterized a cohort of nine patients with LOTS through a detailed clinical and video description. We then reviewed the available literature regarding the clinical description of patients with LOTS. Our findings were summarized based on the predominant phenotype of presentation to highlight diagnostic clues to guide the diagnosis of LOTS for different neurology specialists (neuromuscular, movement disorders) and psychiatrist.

**Results:**

We described a cohort of 9 new patients with LOTS seen at our clinic. Our literature review identified 76 patients mainly presenting with a neuromuscular, cerebellar, psychiatric, stuttering, or movement disorder phenotype. Diagnostic tips, such as the triceps sign, distinct speech patterns, early psychiatric presentation and impulsivity, as well as neurological symptoms (cerebellar or neuromuscular) in patients with a prominent psychiatric presentation, are described.

**Discussion:**

Specific diagnostics clues can help neurologists and psychiatrists in the early diagnosis of LOTS disease. Our work also represent the first video presentation of a cohort of patients with LOTS that can help different specialists to familiarize with these features and improve diagnostic outcomes.

**Highlights:**

Late-Onset Tay-Sachs (LOTS) disease, a severe progressive neurological condition, has multifaced presentations causing diagnostic delays that can significantly affect research outcomes now that clinical trials are available. We highlight useful diagnostic clues from our cohort (including the first video representation of a LOTS cohort) and comprehensive literature review.

## Introduction

In 1881 Dr. Warren Tay first described a severe early onset neurodegenerative disease associated with the appearance of a “cherry red spot” in the retina of infants with Ashkenazi Jewish (AJ) ancestry. In 1887, Bernard Sachs named it “amaurotic family idiocy”. It was only in 1910 that accumulation of a “lipoid substances” in the ganglionic cells was noticed associated with a “peculiar balloon-like swelling of the dendrites” [1]. This disorder was later renamed Tay-Sachs disease (TSD) and it was found to be a result of the deficiency of the isoform A of the hexosaminidase enzyme (HEXA) [2]. The gene encoding this enzyme, *HEXA*, was identified in the late 1980s [3,4]. In the same years, later onset forms of HEXA deficiency were described in older infants and children, leading to the characterization of various sub-types of TSD: “late infantile”, “juvenile” and “late onset” [5,6]. GM_2_ gangliosidoses are caused by a deficiency of the beta-hexosaminidase enzyme, a dimeric enzyme consisting of co-enzymes HEXA and HEXB, critical to the degradation of GM_2_ gangliosides. Mutations in either co-enzymes can lead to progressive neurologic degeneration due to accumulation of the GM_2_ ganglioside in the lysosomes of neurons [7]. TSD is caused by biallelic mutations of *HEXA* on chromosome 15. More than 170 pathogenic variants of *HEXA* have been described so far [8]. “Milder” variants (G269S or W474C) are usually associated with later onset forms while other genetic variants (i.e. c.1278insTATC, c.1073+1G3>A, R499H) are responsible for earlier disease onset and severe phenotypes [9]. The disease is highly prevalent among the AJ population, where the carrier frequency is 1 in 31 people [10].

The clinical phenotype of the classical form of TSD presents usually after the first 6 months of life and rapidly progresses. Symptoms are limited to the central nervous system, manifesting with pyramidal signs, seizures and psychomotor regression with death by the age of 5 years. In the juvenile form, patients can survive into the second decade, while the late onset phenotypes is usually characterized by onset in the second or third decade. Although leucocyte HEXA activity is reduced in older patients, this is usually higher compared to the levels reported in the infants affected with the classical TSD. The juvenile and adult onset forms – collectively termed late onset Tay-Sachs disease (LOTS) – are much less well known to clinicians. Adult patients are frequently diagnosed very late in the course of the disease, typically after several misdiagnoses [11]. Depending on the predominant symptoms at onset, LOTS patients will be usually initially assessed either by Movement Disorders or Neuromuscular specialists, or by psychiatrists.

It is important for clinicians to recognize LOTS to shorten the diagnostic journey for these patients, avoid expensive and invasive diagnostic tests (i.e. muscle biopsy, lumbar puncture, etc.), and provide proper monitoring and surveillance for the patient (i.e. screen for co-morbid psychiatric disorders). In addition, there is significant ongoing investigations to find disease modifying therapies for all forms of TSD [12]. The earlier the recognition of the disease and the higher the chance of preventing further irreversible neuronal degeneration, once treatments will be available.

Here, we will present a case series of nine patients affected with LOTS and include a video presentation of the most characteristic features. We will offer practical diagnostic tips to help clinicians recognize this rare but distinctive disorder.

## Methods

We collected and characterized a population of LOTS cases. Through chart review, we noted their demographic data, family history, clinical features (including age of onset, age at diagnosis, first symptoms, past medical and surgical history), genetic and metabolic tests, brain imaging and physical examination. Patients were also videotaped after informed consent was obtained.

Literature reviewed included Pubmed search of articles up to June 2022, utilizing combinations of the following searching terms: “Late-Onset Tay-Sachs”, “LOTS”, “Tay-Sachs”, “HEXA”, “hexosaminidase”, “amaurotic family idiocy”, “GM_2_ gangliosidoses”, “late onset”, “juvenile”, “neuromuscular”, “motoneuron”, “psychiatric”, “psychosis”, “cerebellar”, “ataxia”, “stuttering”, “dystonia”, parkinsonism”, “movement disorder”. Clinical and demographic traits of the subjects described in the literature were collected by the authors and summarized in Table 1 and 2. Only articles available in English were considered.

**Table 1:** **Phenotypic characterization of our cohort.**


		1	2	3		5	6	7	8	9

**DEMOGRAPHYIC INFORMATION**	GENDER	F	F	F	M	F	M	M	F	F

	AGE CLINICAL PRESENTATION	childhood	childhood	NA	childhood	27	25	20	childhood	23

	AGE AT DIAGNOSIS	22	34	27	25	54	47	31	26	prenatal

	ETHNICITY	AJ	AJ	NA	NA	AJ	AJ	AJ	NA	NA

	FAMILY HISTORY	+	+(?)	NA	NA	–	+(?)	–	+	NA

**GENOTYPE**		c.1274_1277dupT ATC/805G>A (p.Tyr427llefs*5/G 269S)	C.805GS>A (pG269S)/ c.1421+1G>C	NA	NA	c.1274_1277dupTAT C/805G>A (p.Tyr427llefs*5/G26 9S)	c.1274_1277dupTAT C/805G>A (p.Tyr4271llefs*5/G26 9S)	c. 1274_1277dupTAT C/805G>A (p.Tyr427llefs*5/G26 9S)	R178H/c.805G>A(P.G269S)	c.1274_1277dupT ATC/805G>A (p.Tyr4271llefs*5/G 269S)

**HEXA ACTIVTTY**	LEUCOCITES	8.4	2%, 4%**	NA	↓	↓	5%	5.60%	NA	NA

	PLASMA	28	NA	NA	0%	NA	NA	4%	NA	2%

**FIRST SYMPTOMS**		slurred speech	falls, slurred speech	NA	leg weakness	leg weakness	leg weakness	jaundice, speech problem	action tremor	psychiatric symptoms, slurred speech

**FIRST DIAGNOSIS**		NA	NA	NA	NA	Muscular distrophy, ALS, SMA	NA	NA	SMA	prenatal diagnosis

**CEREBELLAR** **SYMPTOMS**	GAIT (ATAXIC)	+	+	+	+	+	Wheelchair	+	+	+

	TREMOR	+	+ (I)	+ (I)	+ (P)	+ (P)	+ (R,P, K)	+ (I)	+(R,P)	+ (I)

	DYSMETRIA	+	+	+	+	+	+	+	+	+

	BALANCE	off	off	off	off	off	off	off	off	off

**NEUROMUSCULAR** **SYMPTOMS**	RIGIDITY	–	–	–	–	–	–	–	–	–

	SPASTICITY	–	–	–	–	–	–	–	–	–

	REFLEXES	↑	↑	↑	normal	↑	↑ (BC), ↓ (TC, QC)	↓	↑	↓ UULL, ↑ QC

	PYRAMIDAL SIGNS	–	–	–	–	+	–	–	Babinsky	–

	CORTICAL SIGNS	–	+	–	–	+	+	–	–	–

	NEUROPATHY	–	–	–	–	motor axonopathy	–	LMN active and chronic neuropathy	–	–

	WEAKNESS	+	+	+	+	+	+	+	+	+

	WEAKNESS DISTRIBUTION	TC, IP	TC, IP	TC, IP, QC	TC, IP, QC	proximal UL >UL	TC, IP, QC, TA	TC	TC, IP, QC	TC, IP

	CRAMPS	–	–	+	–	–	–	–	–	–

	FASCICULATIONS	–	–	–	–	–	–	–	–	–

**MOVEMENT** **DISORDERS**	BRADYKINESIA	–	–	–	decrement	–	–	–	–	LUE

	RESTING TREMOR	–	–	–	–	–	+	–	+	–

**OTHER SYMPTOMS**	EYE MOVEMENTS	normal	jerky slow pursuit	jerky saccades	jerky saccades	jerky saccades	jerky saccades, horizontal endgaze ny	jerky saccades	jerky saccades	saccadic intrusions

	SPEECH	slurred and fast	slurred and fast	slurred and fast	dysarthria	slurred and fast	dysarthria (mild)	dysarthria (ataxic)	dysarthria (ataxic)	dysarthria

	DYSPHAGIA	+ (food)	+ (food/liquid)	+ (food)	+ (fluid)	+	+ (food)	NA	–	–

	PSYCHIATRIC SYMTPOMS	–	BP, ADHD	ADHD	depression	–	BP, ADHD	ADD	anxiety	BP, anxiety, ADHD

	COGNITION	language impairment (word finding)	language impairment, forgetfulness	NA	NA	normal	poor memory (MOCA 19/25)	attention deficit	normal	normal

	FALLS	+	+	–	+	+	–	+	+	+

	LOSS OF AMBULATION	–	–	–	–	–	+	–	–	–

**BRAIN MRI**		normal	cerebellar atrophy	NA	NA	cerebellar atrophy	cerebral atrophy	cerebellar atrophy (mild)	cerebellar atrophy (superior vermis)	cerebellar atrophy (mild)


Demographic and clinical features of individuals described in this manuscripts (case 1–9) are reported in the table. ADD: attention deficit disorder; AJ: Ashkenazi Jews; ALS: amyotrophic lateral sclerosis; BP: bipolar; IP: iliopsoas; F: female; K: kinetic; NA: not available; ; LL: lower limb; LMN: lower motor neuron; M: male; MOCA: MOntreal Cognitive Assessment; ny: nystagmus; NA: not available; P: postural; QC: quadriceps; R: rest; SMA: spinal muscular atrophy; TA: tibialis anterior; TC: triceps; UL: upper limb; yo: year old. ** Two separate tests.

**Table 2:** **Phenotypic and biochemical characteristic of LOTS patients reported in the literature.** Cases are grouped according to their main phenotypes. AJ: Ashkenazy Jew; F: females; M: males.


	INITIAL DIAGNOSIS/MAIN PHENOTYPE			

	NEUROMUSCULAR	CEREBELLAR	PSYCHIATRIC	STUTTER

***Number Of Patients***	42	17	10	6

***Age of onset (mean, years)***	17.3	14.2	15.94	7

***Age at diagnosis (mean, years)***	33	33.86	26.34	15.6

***Average Of Delay (mean, years)***	10.05	16.09	11.42	10.5

***M:F***	19:17	8:9	6:3	1:5

***AJ(%)***	51.3%	64.7%	57.1%	33%

***Positive Family History (%)***	58.8%	75%	375%	83.3%

***B-hexosaminidase A activity LEUCOCYTES (mean)***	4.8%	5.96%	7.3%	3%

***B-hexosaminidase A activity PLASMA (mean)***	7.30%	3.24%	6.95%	2%


## Results

### General Description of our cohort

We characterized a series of 9 patients with genetically and biochemically confirmed diagnosis of LOTS who were examined at our clinic (Table 1). The average age of the initial presenting symptoms that led patients to seek medical attention was 26.5 years (18–30 years). The mean age at diagnosis was 38.4 years (22–54 years), yielding an 11.9 year delay in diagnosis, which is typical for patients with LOTS [11]. The most common misdiagnosis was neuromuscular disorders in 4/9 of the cases. A cerebellar syndrome was initially suspected in one patient and psychiatric disorders in two cases. Global cerebellar atrophy was present in 6 patients at brain MRI (in 2 cases imaging studies were not available and in one case brain MRI was normal), with no specific pattern or distribution in discrete portions of the cerebellum. Few patients had a family history of LOTS or other nonspecific neurological diagnoses, such as “gait abnormalities” or muscle disease, in deceased relatives.

In retrospect, almost all patients acknowledged symptoms that could be attributable to LOTS early in their childhood. In particular, six patients reported slurred speech that required speech therapy during their childhood (patients 1, 2, 4, 5, 7, 9) and four cases also presented clumsiness in their young age (patients 1, 2, 4, 7). The speech impairment documented in our cohort was characterized by a pressured, accelerated, and dysarthric speech (Video 1). The clarity of the speech in these patients significantly improved as they voluntarily slowed down the speed of their speech. Language (mostly word finding), memory and attention deficits (as appreciated during the clinical examination) were present in 4/9 subjects in our cohort with 2/9 subjects carrying a specific diagnosis of ADHD, 3/9 patients were diagnosed with bipolar disorders, while anxiety was present in 2/9 patients and depression in one. The majority of the patients had frequent falls (7/9 subjects). Less common symptoms in our series were resting tremor (2/9 cases), cortical signs (3/9 cases), peripheral neuropathy (3/9 cases), and slow saccades. Only one patient lost his ability to walk independently by the age of 26. Patients from our cohort did not report any recurrent comorbid common medical conditions. Two patients received a diagnosis before the onset of the symptoms. Patient 9 received the diagnosis in the context of a prenatal screening and patient 4 was diagnosed after her brother had genetic confirmation of LOTS.

### LOTS phenotypes and misdiagnosis

#### LOTS and neuromuscular disease

In our cohort, four patients were initially diagnosed with a possible neuromuscular or motoneuron (MN) disease (Table 1). *Patient 5* was a 61 year old woman of AJ ancestry who developed weakness in her lower limbs in her 20s, particularly ascending stairs. Electromyography/nerve conduction study (EMG/NCS) demonstrated an axonal neuropathy that led to an initial diagnosis of Amyotrophic Lateral Sclerosis (ALS) or Spinal Muscular Atrophy (SMA) and subsequently of muscular dystrophy. In the following years her gait worsened and she developed slurred speech. At age 54, enzymatic testing eventually confirmed the diagnosis of LOTS. *Patient 3* was a non-AJ, 43-year-old woman who presented with bilateral leg weakness in her 20s. She was initially diagnosed with Lyme disease. She received the final diagnosis of LOTS seven years later. *Patient 7* was a 36-year-old AJ-man who developed difficulties with transfers and frequent falls in his early 20s. He was evaluated only at the age of 29. An EMG/NCV showed neuropathy with active and chronic denervation and he was referred to an ALS center. LOTS was diagnosed at age of 31. In retrospect, he had transitory jaundice right after birth and slurred speech since the age of 3, requiring speech therapy. Although he played numerous sports as a child, he was clumsier compared to his peers. *Patient 8* was a 30-year-old, AJ-woman with a kinetic tremor of the hands since her first decade. At age of 18 she was diagnosed with essential tremor, treated with clonazepam and propranolol with partial benefit. Her exam was notable for proximal weakness. An EMG/NCV showed a chronic motor neuronopathy mostly affecting her lower limbs. A nerve biopsy showed severe, chronic, neurogenic changes. Late onset SMA was diagnosed. She then developed cerebellar signs, including ataxic speech, dysmetria and wide based gait, but it was only at age of 26 that she received a diagnosis of LOTS.

**Figure 1:**
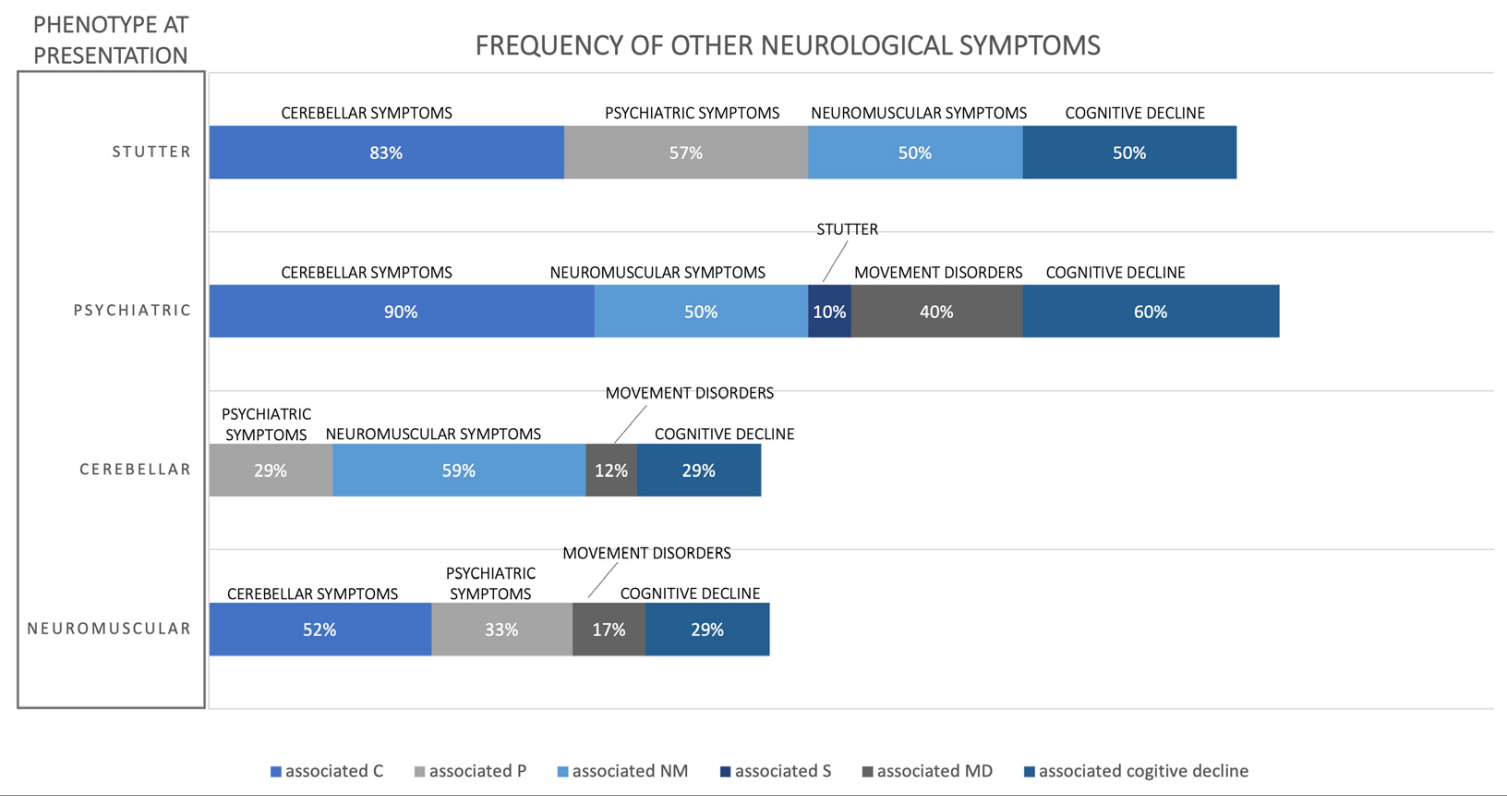
**Signs and symptoms described in patients with LOTS reported in the literature.** Patients are grouped according to the main phenotypes they presented (Neuromuscular, psychiatric, cerebellar, stuttering). Colored bars indicate the frequency of the other symptoms in patients in each category. NM: neuromuscular; MD: movement disorder; P: psychiatric; S: stutter; C: cerebellar. Movement Disorders included dystonia, parkinsonism, chorea, dyskinesia. A contribution to the use of neuroleptics for the treatment of psychiatric symptoms cannot be excluded.

In the literature, 42 single case reports of LOTS presented a prominent motoneuronal phenotype [13,14,15,16,17,18,19,20,21,22,23,24,25,26,27,28] (Table 2). Similarly to our cases, the age of onset was mostly in the second decade, except for few exceptions [21,23,24,25]. The diagnosis was delayed by about 10 years from the age of onset, in line with other cases of LOTS, with an almost equal male/female ratio. Gly269Ser, the most common variant of *HEXA* reported in patient with LOTS, was present in almost all the cases of this cohort where genotype was tested. About half of these patients were of AJ ancestry, although ethnicity was not reported in all published cases. Different from the classical form of ALS, where the onset of disease is heralded by asymmetric and distal atrophy and weakness, the majority of LOTS patients presented symmetric proximal weakness (brachial triceps and thigh quadriceps), with prominent involvement of the lower limbs. In adjunct, these cases still exhibited cerebellar and psychiatric symptoms as well (Figure 1, Video 1). The “triceps sign” is a distinctive features of LOTS and consists in a selective weakness of forearm extension and loss of triceps reflex, with retained forearm flexion and the other reflexes in the upper limbs. Cognitive impairment, when present, was typically mild and usually affecting memory. Differently from the classical motor neuron diseases, ocular movements were variably impaired as well as other bulbar functions. No systemic symptoms or significant family history were reported. Brain MRI always showed cerebellar atrophy, irrespective of clear cerebellar signs. Moreover, in most patients, tremor and ataxic gait presented many years after the initial onset of the symptoms, usually in their 30s [11]. In this cohort of patients atypical features have been reported, such as mild chorea, progressive supranuclear gaze palsy, bilateral cranial VI nerve paralysis, stimulus sensitive myoclonus, bradykinesia, dystonia and dyskinesia, as well as sensory impairment [13,14,17,19,21,24,28]. Four cases presented almost pure MN phenotypes, with involvement of both the upper and lower MN [23]. In all these cases stuttering was described, while only one cases was diagnosed with psychotic symptoms, and one case also presented resting tremor [23].

**Video 1:** **Clinical features of our cohort of patients with LOTS.** Classical clinical signs and symptoms are grouped in sections (“weakness”; “Tremor, dysmetria, and other motor symptoms”, “gait”; “speech”, “eye movements”) where representative clinical exam of some of the patients in our cohort are reported. The age of each subject at the time of the examination is reported in the video. “Weakness”: in this section the clinical exam of patient 2 and patient 6 show a clear pattern of distribution of the weakness involving the proximal extensor muscles (triceps brachii) in the upper limbs, with presentation of the strength of the other muscles in the upper limbs, and weakness of the proximal flexor muscles in the lower limbs (iliopsoas) in subject 1. “Tremor, dysmetria, and other motor symptoms”: this section shows the presence of a distal, postural tremor in the upper limbs and dysmetria of the upper limbs in patient 2 and patient 5, as well as increased deep tendon reflexes of the biceps brachii, and crossed adductor reflex in patient 2. “Gait”: gait impairment due to cerebellar features and lower limbs weakness. Gait in subject 1 showed a mild narrowing of the base and significant difficulties with tandem gait. Patient 3 and 5 show a wide base gait and instability due to ataxia and proximal weakness. “Speech”: speech in subjects 1, 2, and 6 presents the classical features of a pressured dysarthric speech, which is characteristically heard in LOTS. “Eye movements”: eye movement exam in patient 1 and 6 shows the present of saccadic intrusion of the pursuits and overshooting of saccadic movements due to a cerebellar involvement.

In the literature different studies screened populations of patients with pure MND but failed to identify mutations of the *HEXA* gene [29,30,31]. The presence of atypical features (i.e. associated cerebellar signs), and/or predominant proximal weakness (triceps and quadriceps weakness) should prompt adding LOTS in the list of differential diagnosis in these cases.

#### LOTS and psychiatric abnormalities

Psychiatric presentations are common among patients with LOTS. A seminal work by Navon et al. calculated an incidence of 28% of psychiatric features in patients with LOTS [23]. They identified two major phenotypes: depression with psychotic features, and disorganized schizophrenia, both associated with different degrees of cognitive deterioration [23]. However, later reports indicated an incidence of psychiatric features in up to 50% of cases [32,33]. In our cohort, three cases presented with prominent psychiatric manifestations. *Patient 6* is a 52-year-old, AJ-man who presented with a psychotic episode at age 27. In retrospect, he noted weakness in his lower limbs since the age of 25 manifesting as difficulty shifting his legs while driving a bus, as well as difficulties focusing since he was in school. His motor symptoms progressed but a diagnosis of LOTS was confirmed much later, at age 47. *Patient 9* is a 23-year-old, AJ-woman initially diagnosed with LOTS during a prenatal screening. At age 23, she had a manic episode characterized by decreased sleep, grandiosity, depersonalization and paranoid delusions that led to a brief psychiatric hospitalization. She was treated with lithium, carbamazepine and clonazepam and was able to return to college. However, she was already exhibiting slurred speech (requiring speech therapy), cerebellar signs, and proximal muscle weakness. Looking back, since high school she had bilateral leg weakness and balance impairment, with difficulties keeping up the pace when walking with peers and tended away from athletic activities. Similarly, *patient 2* presented in her early twenties a maniac episode characterized by sleep deprivation, impulsive money spending, increased sexual activities with promiscuity that led to the diagnosis of bipolar disorder, treated with aripiprazole, fluoxetine, and bupropion. LOTS was diagnosed only a decade later due to the concomitant cerebellar symptoms (as described below). In addition to mood disorders and psychosis, a significant number of case in our cohort (5 out of 9 cases) also presented attention deficit disorders since their early ages often associated with learning difficulties. Disinhibition was present in 67% of cases as well.

To date, ten cases of LOTS have been reported in the literature with a psychiatric impairment as the first symptom [23,25,28,33,33,34,35,36,37,38] (Table 2). Interestingly, upon further review, some of these patients were already exhibiting other features, such as speech or gait impairment, even before the psychiatric manifestations [25,33,34,35,36]. In almost all the cases psychiatric symptoms became evident in the late teen ages or early 20s.

Seizures preceding psychiatric abnormalities were reported only in one case with onset at 20 years of age, representing the first manifestation of LOTS [35]. Auditory hallucinations have been reported as well [22]. One report described a woman who experienced post-partum psychosis, which resolved within one month after treatment with lithium [39].

Based on literature review, there is a significant delay in the diagnosis in patients presenting with predominant psychiatric manifestations (mean years of delay in diagnosis is 11.42 years, Table 2). The reported male to female ratio is 6:3, although the number of patients is still very low to drive conclusive observations (Table 2).

#### LOTS and cerebellar impairment

Although cerebellar atrophy and cerebellar signs are well described in both TSD and in LOTS, few cases have been reported as pure cerebellar syndromes and yet those are still often misdiagnosed for other forms of inherited ataxia syndromes. In line with this observation, in our cohort we identified only one patient presenting with prominent cerebellar features. *Patient 2* is 40-year-old, AJ-woman who started presenting progressive gait and balance impairment as well as slurred speech and dysphagia since childhood. These symptoms were initially attributed to the sequelae of a presumed cerebral palsy. At age 25 she was diagnosed with bipolar disorder. A brain MRI at age 30 showed cerebellar atrophy and spinocerebellar atrophy (SCA) was suspected. The rapid progression of her symptoms prompted further diagnostic workup and a reduced HEXA activity was detected. Genetic analysis confirmed a compound heterozygote variant in the *HEXA* gene (c.805G>A/c.1421+1G>C).

Seventeen cases have been reported in the literature were patients were initially diagnosed with Friedreich ataxia, spinocerebellar ataxia or tremor [25,28,40,41,42] (Table 2). In these cases age of onset was mostly in the first decade, therefore slightly earlier compared to patients presenting with MND or psychiatric symptoms, with only few cases with onset in the second or third decades [25,28]. In one of these cases the symptomatology presented as an isolated action and resting tremor, and as tremor associated with psychotic symptoms in the other one [25]. There was an equal distribution between males and females. The delay of diagnosis is much longer compared to the other phenotypes (16.1 years on average). 64.7% of the cases were of AJ ancestry. Only 29% of the cases presented initially with psychiatric features, while almost half of the cerebellar cases also had pes cavus, which is interestingly is more frequently associated with Sandoff disease.

#### LOTS and stuttering

Interestingly in a small subset of cases in the literature (2 reports, describing a total of 6 cases) LOTS mainly manifested with stuttering of the speech [43,44] (Table 2). Speech abnormalities are frequently a very prodromal symptom of LOTS, although most of the time they go undiagnosed because of the very low specificity of the symptoms (Video 1). In most of the reported cases presenting with stuttering, this symptom presented during elementary school. In only one case the symptoms manifested when the patient was 16 years old, associated with dysarthria [44]. Females seem to be affected by this phenotype five time more than males, although the overall population is very small. Those cases are mostly European and with no AJ ancestry. The delay in diagnosis range from 3 to 16 years. Interestingly, all the cases presented with a similar timeline: stuttering presented in the first decade followed by weakness and clumsiness in adolescence (in 2 patients) and then psychiatric impairment in their twenties. Ataxia, dysarthria and pyramidal signs presented later. Tremor was present only in a minority of the cases. These patients did not reported other characteristic features.

Interestingly, other late-onset forms of lysosomal storage disorders may also present with stutter, especially GM1 gangliosidosis, metachromatic leukodystrophy, Krabbe disease and Niemann–Pick type C, all of which have overlapping clinical features with LOTS [43]. The possible correlation between lysosomal storage disorders and stuttering is not clear yet.

None of our patients specifically stated that they had stuttering as part of their speech impairment either now or in retrospect. Their speech was characterized as ataxic dysarthria, however, stuttering was apparent on exam in 3 of them.

#### Other features

Other common features of LOTS are usually identified after the diagnosis is made. Eye movement abnormalities have been extensively described and analyzed in this disorder consisting in a disruption of the normal saccadic system, with hypometria, transient decelerations, and premature termination [45] (Video 1). Nystagmus and impaired saccades have been reported in 21% of the cases where eye movements were assessed [17,18,33,40,41,46]. Loss of vertical eye movements has been described in 8 cases in the literature, often incomplete and affecting the upbeat gaze [17,25,36,40,46,47,48]. The presence of supranuclear ophthalmoplegia is associated with a very wide age of onset and with either ataxic, psychiatric or neuromuscular impairments at presentation.

Another recurrent features in LOTS is the presence of pes cavus, not necessarily associated with the neuromuscular phenotype [25,40,41,49]. While Shapiro et al. described a prevalence of 27% of axonal peripheral neuropathy in a cohort of thirty patients with LOTS, this feature has not been frequently observed in the clinical evaluation of the cases reported in the literature, where only two cases presented sensory symptoms, and other two were diagnosed with peripheral neuropathy [19,24,42,48,50].

#### Parkinsonism/basal ganglia phenotypes

Different movement disorders have been associated with LOTS in the literature. In the cohort described by Argov et al. (1984), one patients initially presented with a neuromuscular phenotype during adolescence, then complicated by cerebellar signs and tremor [25]. After two decades from the onset he developed rigidity and bradykinesia, with dramatic response to levodopa but early manifestation of motor fluctuations and dyskinesia, after only two years [51]. In the same paper, the father of one patient, who had intermediate deficiency of hexosaminidase activity, developed Parkinson’s disease at age 45, and 20 years later developed severe cognitive impairment [25]. Autoptic cases of patients diagnosed with LOTS showed different degrees of degeneration of the basal ganglia, particularly in the substantia nigra [52].

Dystonia can be part of LOTS but has never been described as an isolated feature [28,35,37,49,53]. In these cases, dystonia usually involves the limbs, but facial grimace or speech impairment have been reported as well [21,40,46]. Chorea and stimulus sensitive myoclonus were reported in few isolated cases [13,19,48,54].

## Discussion

Classical TSD is a devastating neurodegenerative disorders, leading to death within the first few years of life and is easily recognized. However, the existence of attenuated forms of TSD, which can present in the first decade in life or later, prove to be a diagnostic challenge for both patients and clinicians [55]. Patients with LOTS manifest a wide spectrum of phenotypes, resembling motor neuron diseases, cerebellar degeneration, and/or psychiatric disorders. A large, previously described cohort of patients defined the natural history of this disorder [11]. Due to its rarity and heterogenous presentations, LOTS is frequently undiagnosed or recognized only long after the onset of the symptoms. Because a growing number of potential disease modifying therapies are under evaluation for LOTS, it is now becoming critical to promptly recognize this disorder.

LOTS should be added to the list of differential diagnosis of anterior horn cell disorders, neuromuscular diseases, or ataxias, as well as in the event of acute onset of psychosis or mood disorders in young adults. A general overview of the reported cases suggest that the onset of the disease may occur between the first and the third decade, although an attentive revision of patient’s clinical history often highlighted the disease’s subtle hallmarks earlier in life. This suggests that maybe the real watershed between the juvenile and late onset TSD is the severity of the progression of the symptoms across the second and third decade rather than the age of onset of the first manifestations. In the majority of the cases speech impairment, with a characteristic accelerated speech, can be present in the earliest school years requiring the assistance of speech therapy. Interestingly, clinical manifestations seem to be consistent among patients in the same family. Cerebellar symptoms usually present earlier (in the first decade), followed by neuromuscular manifestations (second decade), and psychiatric symptoms (second-third decade) [11].

**Figure 2:**
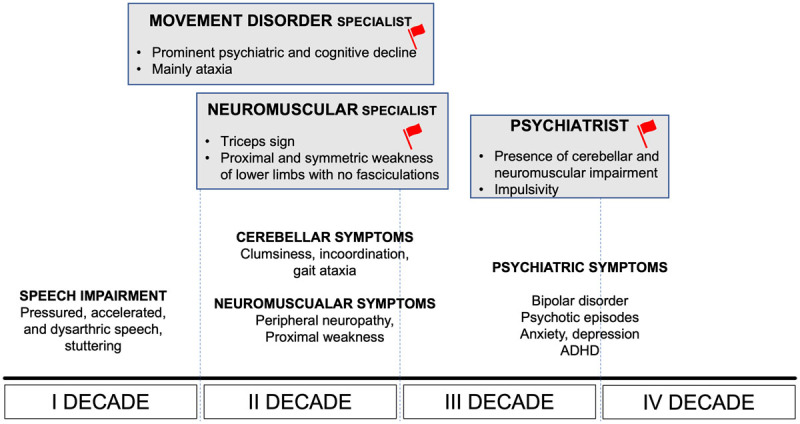
Diagnostic tips for early diagnosis of LOTS according to the possible phenotypic presentation to different neurology specialists and age of onset.

Compared to other lysosomal storage disorders, such as Gaucher disease, there is no report of systemic involvement of this disorder [56]. It is also worth noticing that the typical cherry red spot, usually detected as a characteristic sign of the classical, early onset form of the disease is not present among LOTS patients, who instead manifest eye movement abnormalities, mostly affecting the saccadic phases. Despite the different clinical pictures, brain MRI always consistently shows cerebellar atrophy, even when cerebellar signs are not prominent. In the majority of cases there is a gradual progression of the symptoms, with a step by step involvement of the different systems. Besides considering the broad spectrum of presentations of the disease, some other tips may be helpful to suspect LOTS such as the characteristic proximal weakness (particularly affecting the triceps in the upper limbs), the accelerated speech, a significant impulsivity often leading them to dangerous choice in life or predisposing them to frequent falls when gait is more compromised (Figure 2).

The present work has the limitation of being a retrospective analysis of reported cases. Some of the symptoms may be underestimated due to underreporting rather than absence of the features. However, this reasoned review of the literature, together with the analysis of our cohort of patients and their video presentation will offer a solid base to help clinicians identify LOTS earlier thereby preventing unnecessary tests and diagnostic assessments, as well as allow better counseling to the patient, and opening the possibility of earlier and more effective therapeutic intervention when it becomes available.
